# Functional analysis of the dihydroflavonol 4-reductase family of *Camellia sinensis*: exploiting key amino acids to reconstruct reduction activity

**DOI:** 10.1093/hr/uhac098

**Published:** 2022-04-22

**Authors:** Haixiang Ruan, Xingxing Shi, Liping Gao, Arif Rashid, Yan Li, Ting Lei, Xinlong Dai, Tao Xia, Yunsheng Wang

**Affiliations:** 1 State Key Laboratory of Tea Plant Biology and Utilization, Anhui Agricultural University, Hefei, Anhui 230036, China; 2 School of Life Science, Anhui Agricultural University, Hefei, Anhui 230036, China; 3 College of Tea Science, Guizhou University, Guiyang Guizhou 550025, China

## Abstract

Anthocyanins and proanthocyanidins (PAs) are important types of flavonoids, plant secondary metabolites with a wide range of industrial and pharmaceutical applications. DFR (dihydroflavonol 4-reductase) is a pivotal enzyme that plays an important role in the flavonoid pathway. Here, four *CsDFR* genes were isolated from *Camellia sinensis*, and their overexpression was analyzed *in vitro* and *in vivo.* Based on transcription and metabolic analyses, *CsDFR* expression was closely consistent with catechins and PAs accumulation. Moreover, enzyme activity analyses revealed that the two recombinant proteins CsDFRa and CsDFRc exhibited DFR activity, converting dihydroflavonols into leucoanthocyanins *in vitro*, but CsDFRb1 and CsDFRb3 did not. *CsDFRa* and *CsDFRc* overexpression in *AtDFR* mutants (*tt3*) revealed that *CsDFRs* are involved in the biosynthesis of anthocyanins and PAs, as *CsDFRa* and *CsDFRc* restored not only the purple petiole phenotype but also the seed coat color. Site-directed mutagenesis revealed that the two amino acid residues S117 and T123 of CsDFRa play a prominent role in controlling DFR reductase activity. Enzymatic assays indicated that CsDFRa and CsDFRc exhibited a higher affinity for DHQ and DHK, respectively, whereas CsDFRb1^N120S^ and CsDFRb1^C126T^ exhibited a higher affinity for DHM. Our findings comprehensively characterize the DFRs from *C. sinensis* and shed light on their critical role in metabolic engineering.

## Teaser

We report the first class II/III dihydroflavonol 4-reductase genes (*CsDFR*s) in plants. This finding not only sheds light on *DFR* gene evolution but also provides candidate *DFR* genes for flavonoid metabolic engineering.

## Introduction

Flavonoids, which include flavones, flavanols, flavonols, flavan-3-ols (catechins), anthocyanidins, and proanthocyanidins (PAs), are a class of naturally occurring secondary metabolites that are widely distributed in different plant tissues, such as leaves, flowers, stems, and fruits [1]. Flavonoids are also highly potent and health-promoting compounds. In epidemiological, clinical, and animal studies, they act against different diseases, such as cardiovascular disease, cancer, and other disorders [2–4]. The metabolic and biosynthesis pathways of plant flavonoids have piqued interest in the field of plant secondary metabolites because of their functionalities, compelling evidence of health benefits, industrial applications, and incorporation into various food products.

**Figure 1:**
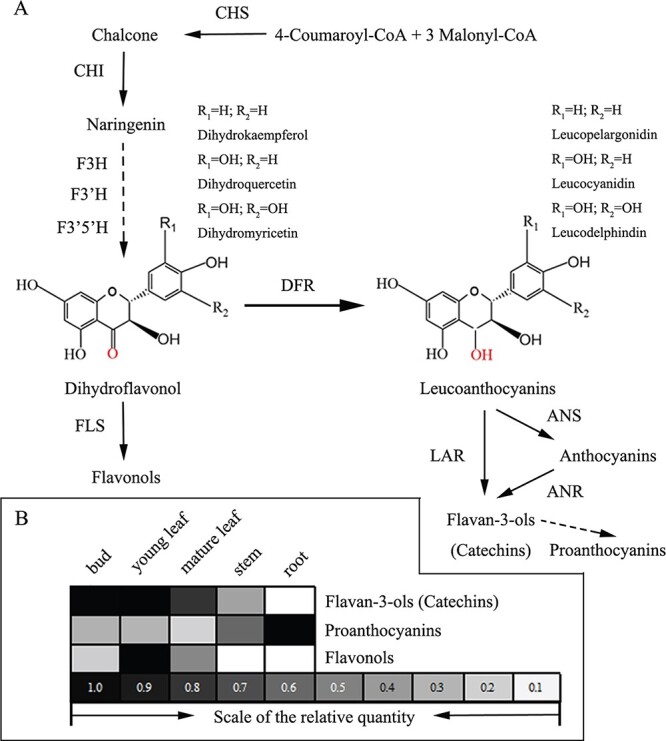
The flavonoid biosynthesis pathway and the end-product accumulation of flavonoids in *C. sinensis.* (A) Flavonoid biosynthesis. CHS, chalcone synthase; CHI, chalcone isomerase; F3H, flavanone 3-hydroxylase; F3′H, flavonoid 3′-hydroxylase; F3′5′H, flavonoid 3′5′-hydroxylase; DFR, dihydroflavonol 4-reductase; FLS, flavonol synthase; LAR, leucoanthocyanidin reductase; ANS, anthocyanidin synthase; ANR, anthocyanidin reductase. (B) The proportions of various flavonoid compounds. The proportions of flavonoid compounds were taken from Jiang [7].

The flavonoid biosynthesis pathway has been extensively investigated in numerous plant species at the genetic, molecular, and biochemical levels, and key functional genes and transposable elements have been identified. Naringenin is an important precursor that can be hydroxylated by flavonoid-3-hydroxylase (F3H) at the 3-position of the C ring or hydroxylated by flavonoid-3′-hydroxylase (F3′H) or flavonoid-3′5′-hydroxylase (F3′5′H) to produce three types of dihydroflavonols, DHK (dihydrokaempferol), DHQ (dihydroquercetin), and DHM (dihydromyricetin) [5]. DFR (dihydroflavonol 4-reductase) is an enzyme that converts dihydroflavonols to leucoanthocyanidins, which are precursors to anthocyanidins, flavan-3-ols, and PAs (Figure 1A) [6].

DFR is a key rate-limiting enzyme of flavonoid biosynthesis that belongs to the NADPH-dependent epimerase/dehydratase family. Genes that encode the DFR enzyme have been characterized and isolated from a variety of plant species, and their functions have been studied extensively [8–11]. Several reports have shown that *DFR* is involved primarily in the color of the petals and seed coat. The *Arabidopsis thaliana* DFR-deficient mutant *tt3*, for example, shows a complete lack of PA accumulation and a transparent seed coat, and DFR-deficient gerbera has a normal phenotype of white-colored petals [12, 13]. Other *DFR*-like genes in plants, such as *OsDFR2* from *Oryza sativa* and *DRL1* from *Arabidopsis,* have been shown to have various biochemical and physiological activities in plants, and they are essential for pollen formation and male fertility.

Several studies have shown that DFRs from different species have distinct substrate specificities, such as the DFRs from *Cymbidium* [14] and *Petunia* [15] that do not effectively reduce DHK, the precursor to orange pelargonidin-type anthocyanins. Previously, a 26-amino-acid region in the middle of the sequence was identified as determining the substrate specificity of DFR proteins [15].

*Camellia sinensis* is a commercially significant plant that produces one of the most popular non-alcoholic beverages on the planet. As well as having a distinct flavor, tea is regarded as a healthy drink owing to its nutritional and therapeutic properties [16, 17]. Tea has become one of the most prominent symbols of Chinese culture. In recent years, the health-promoting properties of tea have been attributed mainly to its polyphenol content, particularly that of flavan-3-ols (catechins) and flavonols, which comprise 30% of fresh leaf dry weight [7, 18]. Catechins with gallic acid esters, such as epigallocatechin-3-gallate (EGCG) and epigallocatechin (ECG), are the most abundant catechins in tea and have received the most attention [19]. However, the total amount of PAs in roots, particularly proanthocyanidin dimers and trimers, is significantly higher than that in leaves and stems (Figure 1B and Table S1) [7]. Overall, these findings reveal that the tea plant accumulates high levels of catechins, but the molecular mechanism underlying catechin metabolism and regulation in tea leaves remains elusive.

**Figure 2:**
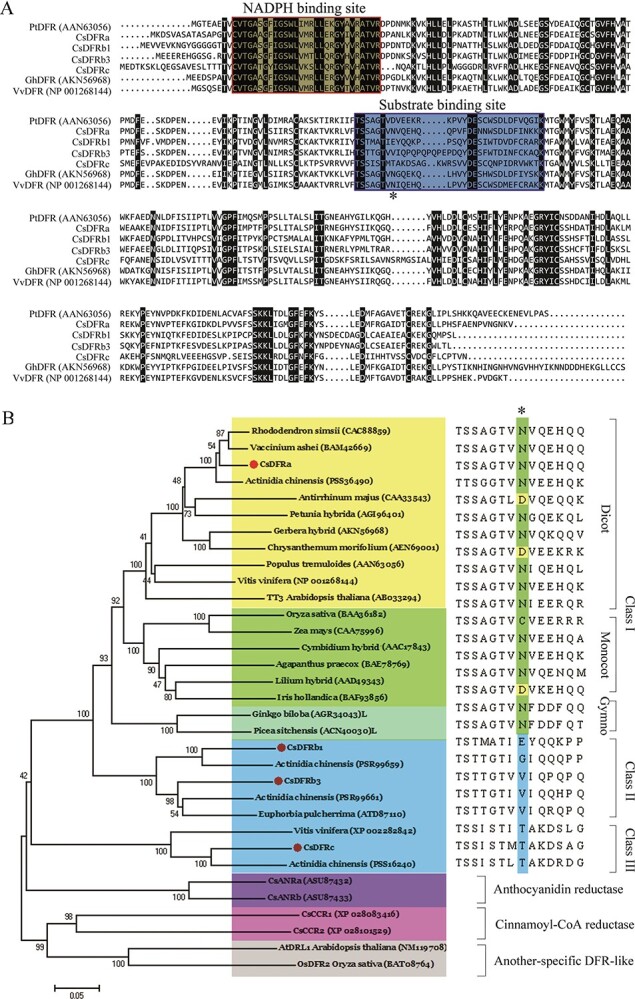
Sequence analysis of CsDFRs. (A) Multiple sequence alignment of DFRs from various plant species. The NADPH-binding region and substrate-binding site are boxed in red and blue, respectively. (B) Phylogenetic tree of CsDFRs and other reductases from different plant species. The accession numbers were obtained from the publicly accessible NCBI database and are presented in the figure.

Previous studies have indicated that *CsDFR*a is a key gene for the regulation of catechin content in tea plants [20]. Previously, five *CsDFR* genes were identified using genome sequences and transcriptome databases [21]. In this study, four *CsDFR* genes (*CsDFRa*, *CsDFRb1*, *CsDFRb3*, and *CsDFRc*) were identified and cloned from the bud/root of *C. sinensis cv. Shuchazao*, and their expression patterns in different tissues were analyzed using qRT-PCR. A phylogenetic analysis revealed that CsDFRa was clustered into class I, and the other three *C. sinensis* proteins were clustered independently, separate from the other NADPH-dependent reductase branches (class II and class III). A prokaryotic expression vector was constructed, and the enzyme activity was determined *in vitro*. To elucidate the functions of CsDFRs *in vivo*, *CsDFRs* were overexpressed in the *Arabidopsis tt3* mutant to observe phenotypic and metabolic changes. To determine which region of the CsDFRa and CsDFRb1 enzymes was responsible for their substrate specificity, we introduced chimeric CsDFRs and determined the activity region. We confirmed that the activity of CsDFR could be modulated by altering the amino acids in this region. This paper comprehensively and systematically reports the functional identification of *CsDFR* class II and class III genes in plants. We propose that these findings will not only aid in the study of *DFR* gene family evolution in shrub/vine plants but will also provide new candidate *DFR* genes for flavonoid metabolic engineering.

## Results

### Cloning and sequence analysis of CsDFRs

Based on the genome sequence and transcriptome information of *C. sinensis,* five putative *CsDFR* genes were screened and identified: *CsDFRa* (GeneBank ID: KY615690), *CsDFRb1* (KY615691), *CsDFRb2* (KY615692), *CsDFRb3* (KY615693), and *CsDFRc* (KY615694) [21]*.* To clone the *CsDFR* genes, cDNA libraries were constructed from the buds and roots of *cv. Shuchazao* and screened using gene-specific primers for *CsDFRs* from *C. sinensis* as listed in Table S2. The other four genes, except for *CsDFRb2,* were successfully cloned and sequenced (Figure S1A). The sequencing results showed that the ORFs of *CsDFRa*, *CsDFRb1*, *CsDFRb3*, and *CsDFRc* are 1044 bp, 1035 bp, 1023 bp, and 1074 bp, respectively, encoding 347, 344, 340, and 357 amino acids (Figure S1A). The characteristics of the four structural genes are shown in Figure S1B. The four *CsDFR* genes have similar genetic structures, with 6 exons and 5 introns, similar to *DFR* genes from other plants such as *Arabidopsis* and buckwheat [22, 23]. To better understand the function of the *CsDFR* genes, we predicted major transcription factor binding motifs in their promoter regions (Figure S1C). MYB binding sites were detected in the promoter regions of *CsDFRa*, *CsDFRb1*, and *CsDFRc*. However, no MYB binding site was detected in the promoter region of *CsDFRb3*, and the MYB motifs MYBPLANT and MYBPZM were not found in the promoter region of *CsDFRb1*.

Sequence alignment with several previously identified DFRs revealed that the N-terminal regions of the *C. sinensis* DFR proteins comprised putative NADPH and substrate binding sites (Figure 2A). Compared with other DFR proteins, the substrate binding regions of the CsDFRb3 and CsDFRc sequences had 5 or 3 redundant residues, respectively. DFR proteins can be classified into three types based on the conservation of the 134th residue: asparagine (Asn), aspartic acid (Asp), and neither Asn nor Asp [15]. CsDFRa, like most DFR proteins from other species, is an Asn-type protein, whereas the other three proteins from *C. sinensis* are neither Asn- nor Asp-type (Figure 2).

A phylogenetic tree was constructed using the neighbor-joining method to further investigate the homology of these four proteins with other known DFRs and NADPH-dependent reductases, such as anthocyanidin reductase (ANR), leucoanthocyanidin reductase (LAR), and another-specific DFR-like enzymes. CsDFRa was clustered into class I with proteins from dicot, monocot, and gymnosperm species. Several class I proteins have been reported in previous literature (Figure 2B). CsDFRa was most similar to proteins from *Actinidia chinensis* (98.86% identity), *Rhododendron simsii*, and *Vaccinium ashei* (89.28%). The other three *C. sinensis* proteins were clustered independently, separate from the other NADPH-dependent reductase branches (class II and III), and CsDFRb1 and CsDFRb3 were slightly closer to the core DFR branch. To the best of our knowledge, the functions of genes in classes II and III have not been confirmed *in vivo* or *in vitro,* and most are from shrubs or vines, especially *A. chinensis*, *Vitis vinifera*, and *Manihot esculenta*. Therefore, CsDFRb1, CsDFRb3, and CsDFRc may have special significance for studying the evolution of the *DFR* gene family. The function of these genes requires further elucidation.

**Figure 3:**
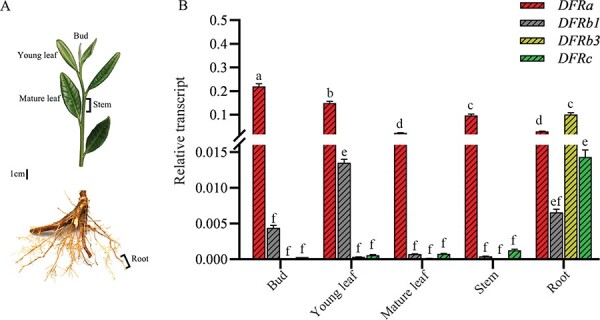
Spatiotemporal expression patterns of *CsDFR* genes in *C. sinensis* based on quantitative RT-PCR analysis. (A) Different tissues collected from numerous plants for gene expression measurements. (B) Transcript levels of the *CsDFRs*. The data are presented as mean ± standard deviation of three biological replicates (n = 3), and GAPDH was used as an internal control for the normalization of the Ct values. Lowercase letters indicate significant differences at *P<*0.05.

**Figure 4:**
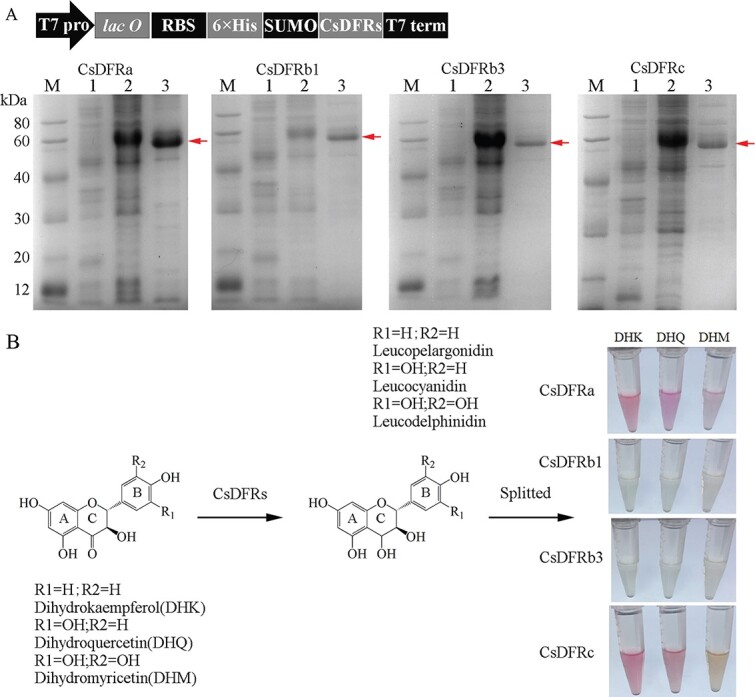
Analyses of catalytic reaction products with CsDFR fusion proteins. (A) Schematic map of the recombinant CsDFR constructs and profiles of expressed CsDFR fusion proteins. M: Protein molecular weight markers; 1: before induction; 2: total protein after induction; 3: purified CsDFR protein. (B) Analysis of the enzyme reactions with SUMO-CsDFRs *in vitro*, and DHK, DHQ, and DHM as the substrates.

### Expression patterns of *CsDFRs* in tea plants

Quantitative RT-PCR was used to detect the expression patterns of *CsDFR* genes in tea plants. Gene-specific primers were used to amplify the four genes from multiple tissues: buds and young leaves, mature leaves, stems, and roots (Figure 3A). The *GAPDH* gene was used as a reference control for normalization. Each gene was differentially expressed in distinct tissues (Figure 3B). For example, transcripts of *CsDFRa* were detected in all tissues examined, with the highest expression level in the bud, followed by the young leaf and stem, and a low expression level in the mature leaf and root. The expression pattern of *CsDFRa* was consistent with the concentration of catechins in tea plants (Figure 1B). However, the expression levels of other genes such as *CsDFRb1*, *CsDFRb3*, and *CsDFRc* were highest in the root and lowest in the mature leaf and stem, consistent with the concentration of PA and indicating the involvement of these three genes in PA biosynthesis in the tea plant. Based on RNA-seq and qRT-PCR results, the expression of *CsDFRa* (mean Ct 19.67 and RPKM 696.54 in the bud) was significantly higher than that of the other three genes (Dataset S1).

### Catalytic activity of recombinant CsDFR proteins *in vitro*

To further analyze the enzymatic properties of CsDFRa, CsDFRb1, CsDFRb3, and CsDFRc, a series of SUMO vectors carrying different *CsDFR* genes were constructed and successfully expressed in *Escherichia coli.* An SDS-PAGE electrophoretogram showed that clear target bands were obtained after IPTG induction of the recombinant strains (Figure 4A).

We detected the anthocyanin product, split from leucoanthocyanidin under high temperature and acidic conditions, because the leucoanthocyanidin produced by the DFR enzyme is highly unstable and difficult to detect directly by HPLC. The red products were observed in the culture of the recombinant strains carrying the *CsDFRa* or *CsDFRc* gene using different substrates: DHK, DHQ, and DHM. As illustrated in Figure 4B, when dihydroflavonols were used as a substrate, no red products were observed, indicating that CsDFRb1 and CsDFRb3 had little or no reduction activity.

### Functional confirmation of the CsDFRs in *Arabidopsis*

*Arabidopsis tt3* is a *DFR* mutant that not only shows a deficiency in anthocyanin synthesis but also exhibits an absence of seed coat pigment. Therefore, the *tt3* mutant was a suitable model plant for determining whether *CsDFR* was associated with the biosynthesis of anthocyanins and PAs.

**Figure 5:**
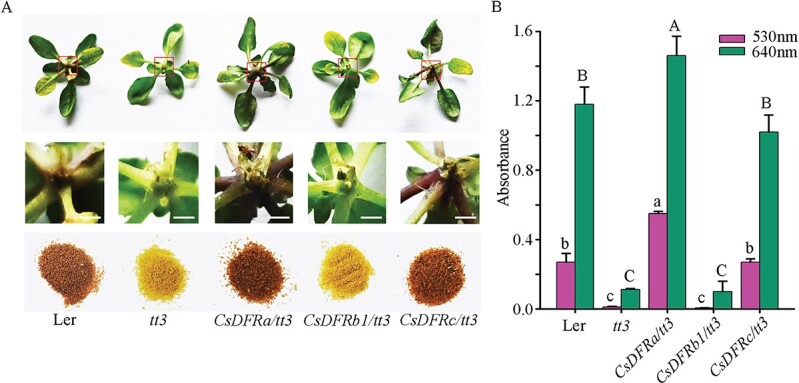
Overexpression of *CsDFRs* in the *Arabidopsis* mutant *tt3*. (A) Phenotypes of Ler, *tt3*, *CsDFRa/tt3*, *CsDFRb1/tt3*, and *CsDFRc/tt3* seedlings or seeds. (B) The absorbance values of the anthocyanin extracts and of bluish compounds from DMACA reactive compounds were measured at 530 nm and 640 nm, respectively. All values are the means of three biological replicates, and the different letters indicate significant differences at *P*<0.05.

The ORFs of three *CsDFRs* (*CsDFRa*, *CsDFRb1*, and *CsDFRc*) were transformed into the *tt3* mutant driven by the 35S promoter to determine whether *CsDFRs* act as functional *tt3* orthologs that can restore the above-mentioned deficient phenotypes of the *Arabidopsis tt3* mutant. After overexpression of *CsDFRs* in the *tt3* mutant, perceptible phenotypic differences were observed in the *CsDFRa* and *CsDFRc* transgenic seedlings and/or seed coats relative to the *tt3* mutant (Figure 5A). *CsDFRa* and *CsDFRc* not only restored the purple petiole phenotype but also recovered the seed coat color of the *tt3* mutant when compared with *tt3* and *CsDFRb1* transgenic linesx (Figure 5A).

DFR converts dihydroflavonols into leucoanthocyanins in the flavonoid biosynthesis pathway. Leucoanthocyanins are then converted into anthocyanins by ANS. These aforementioned compounds play a role in the synthesis of PAs in plants [24, 25]. Polyphenols were extracted from wild type (Ler), *tt3* mutant, and *CsDFR* transgenic lines, and contents of anthocyanins and soluble PAs were detected in the seedlings and seeds. *CsDFRa* and *CsDFRc* transgenic *Arabidopsis* seedlings had significantly higher anthocyanin contents than *CsDFRb1* and *tt3* mutant lines (Figure 5B). Furthermore, DMACA staining revealed that bluish compounds were formed in seed extracts of the *CsDFRa* and *CsDFRc* transgenic lines, indicating that PAs were increased in the seeds compared with the *tt3* mutant control, along with the wild type as a positive control (Figure 5B). These findings suggested that *CsDFRa* and *CsDFRc* were involved in the biosynthesis of anthocyanins and PAs, consistent with the catalytic functions of CsDFRa and CsDFRc, which can catalyze the transformation of dihydroflavonols into leucoanthocyanidins. By contrast, CsDFRb1 did not perform this reaction *in vivo*.

### Identification of a region that determines the difference in activity between CsDFRa and CsDFRb1

**Figure 6:**
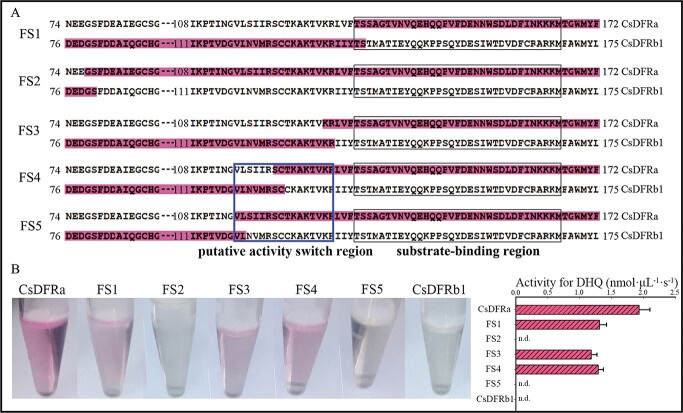
Truncation and reorganization were used to determine the putative activity switch region between CsDFRa and CsDFRb1. (A) Model of amino acid position by truncation. FS1, FS2, FS3, FS4, and FS5 are five different reorganization strategies; amino acid residues with red represent fusion between CsDFRa and CsDFRb1; and the substrate-binding region and putative activity switch region are boxed in black and blue, respectively. (B) Analysis of the enzyme reactions with CsDFRa, FS1, FS2, FS3, FS4, FS5, and CsDFRb1 *in vitro* and DHQ as the substrate.

Although the amino acid sequences of CsDFRb1 and CsDFRa are quite similar, CsDFRa can reduce dihydroflavonol, whereas CsDFRb1 cannot. We used cDNA sequences from these two genes to create five chimeric *DFR* genes in order to identify the region that controls the difference in activity between CsDFRa and CsDFRb1 (Figure 6A and Dataset S2). Prior to catalytic analysis, the proteins were prepared and purified. DHQ was used as the substrate to test the reducing activity of these chimeric DFR proteins (Figure 6B).

The chimeric gene *FS1* was constructed with 5′-*CsDFRb1* and 3′-*CsDFRa* at the beginning of the CsDFRa substrate-binding region, and *FS2* was created with 58 amino acids before the substrate-binding region. The results of activity tests revealed that cyanidin formation was observed in the reaction system with FS1 protein *in vitro* but not in the reaction system with FS2 protein. We speculated that the DFR protein has an activity switch region prior to the substrate-binding region. Based on this speculation, we constructed three more chimeric genes in which the sequence of CsDFRb1 was gradually replaced by that of CsDFRa before the substrate-binding region. Activity tests showed that the FS3 and FS4 recombinant proteins exhibited reductase activity, whereas the FS5 recombinant enzyme had lost its reductase activity. Therefore, we predicted that the amino acid residues between FS4 and FS5 recombination points might have a significant impact on DFR enzyme activity.

### Site-directed mutagenesis of the activity switch region

**Figure 7:**
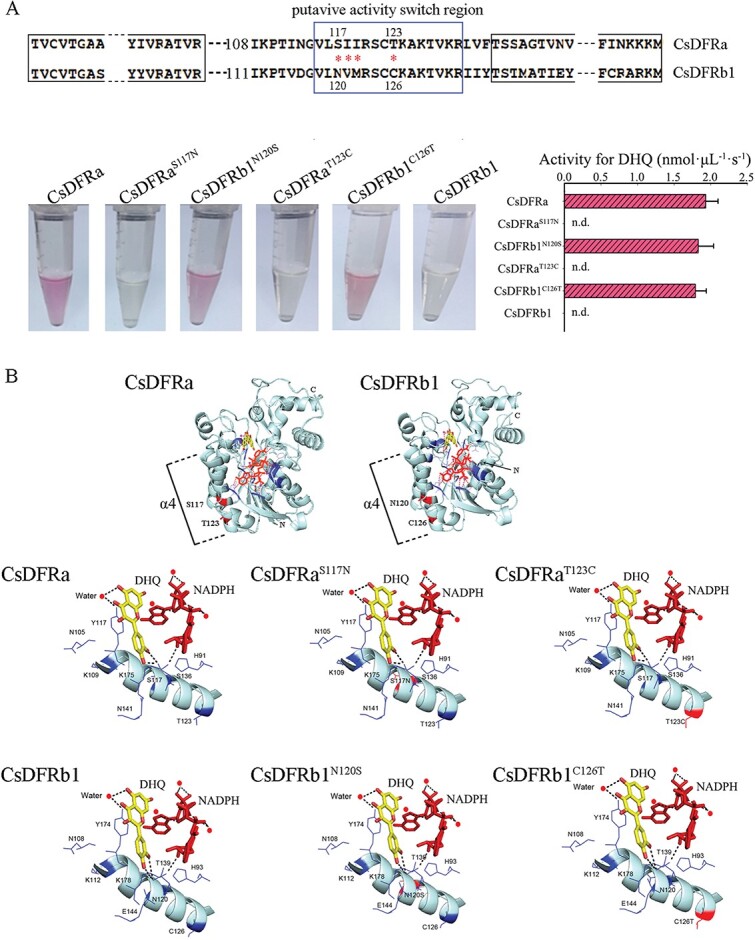
Identification of key amino acids that are critical for the modulation of DFR reducing activity. (A) Analysis of enzyme activities with different site-directed mutagenesis of the activity switch region. The red asterisk indicates the different amino acids of DFRa and DFRb1 at the putative activity switch region. DHQ was used as a substrate to evaluate the reducing activity of the mutated proteins. (B) Homology models of DFRa, DFRb1, and site-directed mutagenesis proteins with docked dihydroquercetin (DHQ) and NADPH ligands; the PAS region is located in helix α4 (red band). The homology model was built using grape DFR crystals as a template (PDB code 3BXX).

An amino acid sequence alignment demonstrated that the amino acids in the putative activity switch (PAS) region of CsDFRa and CSDFRb1 are relatively conserved, with only four different amino acids (Figure 7A). We conducted a series of gene mutation studies on different residues to further confirm that these different amino acids alter DFR activity, and DHQ was used as a substrate to evaluate the reducing activity of the mutated proteins (Figure 7B). The CsDFRa mutated proteins CsDFRa^S117N^ and CsDFRa^T123C^ lost DHQ-reducing activity, whereas DFR activity was restored by the CsDFRb1 mutations CsDFRb1^N120S^ and CsDFRb1^C126T^. Mutations in the other two different residues, such as DFRa^I118V^ and DFRa^I119M^, had no effect on the activity of the non-mutated protein (data not shown). The results of site-directed mutagenesis showed that the serine at 117 and threonine at 123 of CsDFRa are critical for modulating DFR reducing activity. Interestingly, these key amino acids both belong to the hydroxyl amino acid family.

CsDFRa and CsDFRb1 homologous modeling was carried out using dihydroquercetin (DHQ) and NADPH as substrates, and splicing experiments were conducted using grape DFR crystals (PDB code 3BXX) [26]. There were no significant differences in the binding of DFR residues to the substrate and NADPH in the CsDFRs or the site-directed mutated proteins (Figure 7B). Furthermore, the PAS region was located in helix α4, and the X-ray 3D structure revealed that this helix promotes water molecule binding to the carbonyl group of the asparagine main chain and the amino group of the catalytic lysine [27]. This is the first of a cluster of water molecules that transfer a proton from the bulk solvent to the catalytic lysine [28, 29].

### Catalytic activities of recombinant CsDFR proteins *in vitro*

**Figure 8:**
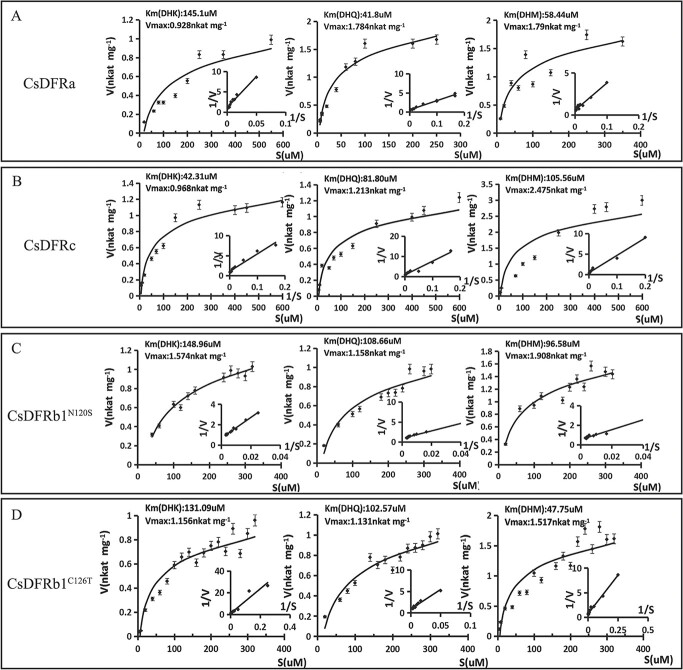
Kinetic parameters of DFRa, DFRc, DFRb1^N120S^, and DFRb1^C126T^. In each enzyme reaction assay, 2 μg of purified recombinant protein was used. A uniform concentration of 0.05 mg dihydroflavonol (DHK, DHQ, and DHM) was used as the substrate. The data are expressed as mean ± SD (n = 3).

The dihydroflavonol substrate kinetic parameters of recombinant CsDFR proteins, including CsDFRa, CsDFRc, CsDFRb1^N120S^, and CsDFRb1^C1267T^, were determined in potassium phosphate buffer at pH 7. CsDFRa *Km* values with DHK, DHQ, and DHM were 145.10, 41.80, and 58.44 μM, respectively. For CsDFRc, the *Km* values for DHK, DHQ, and DHM were 42.31, 81.80, and 105.56 μM, respectively. These results indicate that CsDFRa may have a higher affinity for DHQ, whereas CsDFRc has a higher affinity for DHK (Figure 8 and Table S3).

Enzymatic assays were used to evaluate the activities of the site-directed mutated proteins CsDFRb1^N120S^ and CsDFRb1^C126T^. CsDFRb1^N120S^*Km* values for DHK, DHQ, and DHM were 148.96, 108.66, and 96.58 μM, respectively, whereas CsDFRb1^C126T^*Km* values were 131.09, 102.57, and 47.75 μM. These findings revealed that the N120S and C126T substitutions in CsDFRb1 caused a higher affinity for the DHM substrate (Figure 8 and Table S3).

## Discussion

### CsDFR involvement in the biosynthesis of flavonoids in *C. sinensis*

DFR catalyzes the NADPH-dependent reduction of three different dihydroflavones to form the corresponding leucoanthocyanins [10]. Leucoanthocyanins are the precursors for the synthesis of anthocyanins and flavan-3-ols. Therefore, DFR is the pivotal enzyme responsible for anthocyanin, catechin, and proanthocyanidin accumulation in plants. Catechins, procyanidins, and flavonols are the most abundant flavonoids in *C. sinensis,* with catechins mainly accumulated in buds and young leaves and proanthocyanidins mainly in roots [7, 30].

Numerous studies suggest that *DFR* expression is positively correlated with anthocyanin and PA accumulation. In *Saussurea medusa*, for example, the *SmDFR* gene showed high expression in flowers but significantly lower expression in leaves and roots [31]. In *Lilium asiatica*, two *DFR* genes were highly expressed in the colored parts of the perianth, anther, filament, stigma, and scale [32]. Shimada et al. investigated the tissue expression specificity of *DFR* using *Lotus japonicus* fruit, flower, leaf, stem, and root tissue; they demonstrated that the tissue specificity of DFR gene expression was consistent with its catalytic activity [33]. Tang et al. demonstrated that *AtDFR* was highly expressed during pollen tube development; the results suggested that *AtDFR* may play an important role in pollen maturation and growth [34]. In tea plants, Mamati et al. showed that the *DFR* gene was often highly expressed in tissues with high catechin content [35].

Four *DFR* genes from *C. sinensis* were identified and described in this work. *CsDFRa* was strongly expressed in the bud and young leaf of *C. sinensis*, implying that it is the major gene that determines the accumulation of catechins in the tea plant. The other genes were significantly expressed in the young roots of the tea plant, indicating that these three genes are linked to PAs accumulation in the roots.

### Catalytic specificity analysis of the DFR family

Dihydroflavonols are classified into three types based on the number of hydroxyl groups on the B-ring: DHK (mono-hydroxyl in B-ring), DHQ (di-hydroxyl in B-ring) and DHM (tri-hydroxyl in B-ring). In this study, enzyme activity analysis showed that the four DFR enzymes exhibited different selectivities for the three substrates *in vitro*. According to the protein structure analysis, DFR contains two important domains: the highly conserved N-terminal NADPH binding site and the dihydroflavone substrate binding site [36].

Previous studies have shown that amino acid 134 is important for the catalytic activity of the DFR protein [15]. Therefore, based on the type of amino acid at position 134, DFRs can be divided into three categories. Type 1, called the Asp type, has an aspartic acid (Asp) at position 134 and can catalyze the formation of leucoanthocyanins from DHK. This type of DFR protein has been reported in cranberry [37]. Type 2, called the Asn type, has an asparagine (Asn) at position 134 and cannot catalyze the formation of leucoanthocyanins from DHK. Type 3, called the non-Asp and non-Asn type, has neither an Asp nor an Asn at position 134.

With the development of bioinformatics, *DFRs* have been identified in numerous plant species, and these previously identified *DFR* genes can also be divided into different types. For instance, in *Lotus corniculatus* L., five *DFR* genes can be divided into three types. *LcDFR1* belongs to the non-Asp and non-Asn type, *LcDFR*2 and *LcDFR*3 belong to the Asp type, and *LcDFR*4 and *LcDFR*5 belong to the Asn type [33].

In this study, we found that an understanding of the basic profile of *DFR* involvement in catechin biosynthesis in *C. sinensis* is still being unraveled. An evolutionary analysis showed that *CsDFRa* belongs to Class I, and most genes in this subfamily have been confirmed to have dihydroflavonol reductase activity (Figure 2B). Members of Class I are classified as Asn or Asp types based on differences in the key amino acid sites in the substrate-binding region. A kinetic analysis of recombinant enzymes revealed that CsDFRa had a high DHQ conversion efficiency.

The rooted phylogenetic tree showed that CsDFRb1 and CsDFRb3 were grouped into Class II and CsDFRc was grouped into class III, which belongs to the non-Asp and non-Asn type. The enzymatic activities of members of this subfamily have not been reported previously. Here, we demonstrated that CsDFRc had dihydroflavonol 4-reductase catalytic activity based on functional analysis of prokaryotic recombinant protein and transgenic *Arabidopsis in vitro* and *in vivo*, respectively. Kinetic analysis of the recombinant enzyme showed that CsDFRc displayed the highest conversion efficiency for DHK. However, CsDFRb1 and CsDFRb3 did not exhibit dihydroflavonol 4-reductase catalytic activity.

### Crucial amino acid residues or regions of the DFR protein

Several studies have indicated that DFR substrate specificity is determined by a 26-amino-acid region [15]. CsDFRb1 without catalytic activity and CsDFRa with catalytic activity were truncated and recombined with each other, and the recombinant proteins were expressed in *E. coli* to further investigate the key amino acid residues that determine the function of dihydroflavonol reductase. Analysis of enzyme activity showed that the twenty amino acid residues before the substrate binding region might have important effects on DFR enzyme activity. Furthermore, site-directed mutation confirmed that N120 and T126 are the key amino acid sites that determine the catalytic activity of CsDFRb1. Interestingly, CsDFRb1^N120S^ and CsDFRb1^T126C^ have catalytic activity, and their affinity for DHM is significantly higher than that for DHQ and DHK. These results can be used for the metabolic engineering of B-ring trihydroxyflavonoids.

Homologous modeling and substrate docking showed that site-directed mutation had no effect on the three-dimensional structure of the DFR protein, and there was no significant difference in the spatial conformation between the substrate and NADPH. The putative activity switch (PAS) region is located in the helix α4 region (Figure 7B), which mainly determines the second H^+^ supply in the reduction reaction [27]. Our findings confirmed that the key amino acid residues, serine and cysteine in the PAS region (S117 and C123 of CsDFRa), are mercapto or hydroxyl amino acids that may participate in the transfer of H^+^. The H-bond established by the hydroxyl group with a water molecule or protein is critical for maintaining reductase activity. The establishment of this H-bond cannot be refuted because the binding of a water molecule to the carbonyl group of the main-chain of the asparagine (N120 of CsDFRb1) and the amino group of the catalytic lysine (T126 of CsDFRb1) either breaks the H-bond or induces steric hindrance for substrate binding, which coincides with previous findings [15]. Taken together, these studies provide a theoretical foundation for further study of the catalytic activity of DFR.

## Conclusion

In conclusion, we isolated and characterized the *CsDFR* genes (*CsDFRa*, *CsDFRc*, *CsDFRb1*, and *CsDFRb3*) from *C. sinensis* and demonstrated their roles *in vitro* and *in vivo.* We found that their function is closely associated with catechins and PAs accumulation. Their physiological roles were studied in the *Arabidopsis tt3* mutant, and *CsDFRa* and *CsDFRc* were shown to recover the *tt3* mutant phenotypes. Enzyme activity assays were performed *in vitro* to assess the DFR activity of the CsDFRs, and CsDFRa and CsDFRc had DFR activity that converted dihydroflavonols into leucoanthocyanins. Through site-directed mutations, we found that two amino acid residues of the CsDFRs play an important role in enzyme activity. Enzyme kinetics of recombinant CsDFR enzymes showed that the optimal substrates for CsDFRa and CsDFRc were DHQ and DHK, whereas the optimal substrate for CsDFRb1^N120S^ and CsDFRb1^C126T^ was DHM. Thus, our data suggest that DFRs are evolutionarily conserved in many plant species and help to provide a new candidate gene for the metabolic engineering of flavonoids.

## Methods

### Plant materials

Bud, young leaf, mature leaf, stem, and root tissue of *C. sinensis cv. Shuchazao* were collected in early autumn from a five-year-old plant in an experimental garden at Anhui Agricultural University in Hefei, China (31.86 N, 117.27 E, 20 m above mean sea level). The samples were frozen and stored at −80°C for future use.

Landsberg erecta (Ler, wild type) and the *AtDFR* mutant (*tt3*, CS84) of Ler *Arabidopsis* were obtained from the Arabidopsis Biological Resource Center (abrc.osu.edu) and grown in a growth chamber at the College of Life Sciences, Anhui Agricultural University, with a 16 h light/8 h dark cycle at 22 ± 2°C and a light intensity of 200 μmol m^−2^ s^−1^.

### Isolation and cloning of *CsDFR* genes

The RNAiso Plus for plant tissue kit (Takara, Dalian, China) was used to isolate total RNA from bud and root tissues. Standard end-to-end PCR reactions were performed on the *CsDFR* genes from the NCBI database with gene-specific primers designed according to the cDNA sequence (Table S2). End-to-end PCR was carried out at 98°C for 30 s; 32 cycles at 98°C for 10 s, 58°C for 15 s, 72°C for 30 s; and a final extension at 72°C for 10 min. Subsequently, the PCR amplification products were purified using a Gel Extraction Kit (Aidlab, Beijing, China) and cloned into an Easy-Blunt vector (TransGen Biotech). The resulting vector was introduced into DH5α competent cells and sequenced.

### Phylogenetic analysis

Full-length amino acid sequences of DFR proteins from *C. sinensis* and several other plants were acquired from the NCBI database (https://blast.ncbi.nlm.nih.gov/Blast.cgi) for use in phylogenetic analysis. ClustalW version 2.1 was used to align all of the sequences and deduced amino acid sequences of CsDFRs, and the alignment was submitted to MEGA7 software (https://www.megasoftware.net) to generate a tree with the neighbor-joining method, 1000 bootstrap replicates, and gap handling by complete deletion.

### Quantitative real-time PCR analysis

Gene-specific qRT-PCR primers were designed to study the spatiotemporal expression patterns of *CsDFR* genes in *C. sinensis* (Table S2). Transcript levels were determined using the SYBR premix Ex Taq II kit (Takara, Japan) on a Peltier Thermal Cycler PTC200 (Bio-Rad, USA). All biological samples were analyzed in triplicate. The Ct values of the housekeeping gene glyceraldehyde-3-phosphate dehydrogenase (*GAPDH*) from *C. sinensis* were used to normalize the gene expression levels.

### Heterologous expression of *CsDFRs* in *Arabidopsis*

Using Gateway technology (Invitrogen, USA), full-length *CsDFR* cDNA was shuttled into the pCB2004 binary vector. Subsequently, the positive recombinant pCB2004-CsDFR vector was transferred to *Agrobacterium tumefaciens* (GV3101) using electroporation, and the *Agrobacterium* was then used for floral dip transformation of the *Arabidopsis* mutant *tt3* [38]. The transformed plants were grown in a glasshouse under carefully controlled conditions (16 h light/8 h dark cycle at 22 ± 2°C with 40% relative humidity).

### Extraction and quantification of anthocyanins and PAs

For the extraction of anthocyanins and PAs, freeze-dried samples of seedlings and seeds (0.05 g) from the *CsDFR* transgenic lines, Ler, and *tt3* mutant were ground with a grinder. Subsequently, the total polyphenols were extracted at a low temperature with 1.2 mL of extraction solution A (HCl 0.5%: methanol 80%: water 19.5%, v/v/v), followed by vortexing and sonication for 30 min at 4°C [7]. The samples were centrifuged at 13 000 g for 15 min before being re-extracted twice more as described above until the final volume of the supernatants was 2 mL. Finally, the combined solution was centrifuged for 10 min at 13 000 g, and the supernatants were stored at −20°C prior to analysis of total anthocyanin and PA contents. The total anthocyanin contents in the supernatants were measured with a UV spectrophotometer at 530 nm. After reaction with the DMACA reagent (0.2% DMACA, w/v, HCl/methanol 1:7, v/v), the total soluble PA contents in seeds were determined with a UV spectrophotometer at 640 nm. In brief, the reaction mixture (1.3 mL total volume) contained 0.3 mL of supernatants and 1 mL of DMACA reagent. Subsequently, the reaction mixture was incubated at 25°C for 5 min, and the absorbance values of the reaction mixture were measured at 640 nm.

### Heterologous expression of *CsDFR*s in *E. coli*

Each *CsDFR* ORF was subcloned in its entirety into the pET-SUMO vector (Invitrogen, California, USA). The identity of the cloned gene was validated using T7 primers and sequencing analysis. The resulting SUMO-CsDFR vectors were transformed into *E. coli* BL21 (DE3) competent cells according to the manufacturer’s instructions. All of the chimeric and point-mutated genes were cloned into the pET-SUMO vector by the same method. Positive colonies were cultivated in 200 mL of LB medium with 50 μg·mL^−1^ kanamycin at 37°C. To promote recombinant protein expression, 1 mM isopropyl-β-D-thiogalactoside (IPTG) was added when the *E. coli* OD_600_ reached 0.6. The cells were collected by centrifugation after 8 h of induction at 28°C, and the fusion protein was purified using His-tag affinity chromatography according to the manufacturer’s instructions. Twelve percent SDS polyacrylamide gel staining and Coomassie brilliant blue staining were used to validate the isolated protein fraction. The *in vitro* activity assay was performed using pure recombinant proteins. All specific primers for SUMO-CsDFRs, chimeric CsDFRa/CsDFRb1, and amino acid point mutant genes are listed in Table S2.

### Enzyme activity assay and analysis of kinetic parameters

The enzymatic reaction consisted of 0.1 M pH 7.0 potassium phosphate buffer, 2 mM NADPH, 0.5 mM dihydroflavonols (DHK, DHQ, and DHM) and 0.05 mg total soluble proteins. The reaction mixtures were incubated at 30°C for 30 min, and the total volume was 200 μl. Because leucoanthocyanidin is unstable in solution and difficult to detect directly using HPLC, a 3× volume of butanol–HCl reagent (95:5, v/v) was added, and the reaction was split at 95°C for 1 h to form anthocyanidin, then centrifuged at 4000 g for 15 min. The absorbance of the supernatant was measured at 530 nm with a UV spectrophotometer. Based on the absorbance value of the anthocyanin standard at different concentrations, a standard curve was established to quantify the content of products in the sample. To determine the apparent *Km* value of the reaction mixture with different substrates, 0.05 mg purified recombinant enzyme and three dihydroflavonols at a series of concentrations were used. The enzymatic products were quantified as the standard curve of the anthocyanin standard. Apparent *Km* data were calculated using Hanes plots.

### Molecular docking statistics and site-directed mutagenesis

The homology models of CsDFRa and CsDFRb1 were constructed with the crystal structure of grape DFR (PDB code 3BXX) as a template using the online server SWISS-MODEL (http://swissmodel.expasy.org/interactive). Dihydroquercetin and NADPH were taken as the substrates, and they were docked into the models of CsDFRa and CsDFRb1 with the lowest energy conformation using AutodockTools-1.5.6 (http://mgltools.scripps.edu/downloads). The resulting spatial model structures of the CsDFRa and CsDFRb1 proteins, including binding sites and substrate linkage pockets, were analyzed and visualized using Python 27 software. The site-directed mutation plasmids CsDFRa^S117N^, CsDFRa^T123C^, CsDFRb1^N120S^, CsDFRb1^C127T^, CsDFR^I178V^, and CsDFR^I179M^ were generated using an overlap extension PCR technology with CsDFRa and CsDFRb1 plasmids as the templates. All specific oligonucleotide primers used to construct the mutation plasmids are listed in Table S2.

### Statistical methods

Three separate experiments or three biological replicates were used in each of the aforementioned tests. Mean values were generated and statistically analyzed using Student’s t-test in SPSS software. A two-tailed test of significance was used. P-values of less than 0.05 were considered to be significant.

## Supplementary Material

Web_Material_uhac098

## Data Availability

All data supporting the conclusions of this study may be found in the publication and its supplemental materials, which are available online. Any additional relevant information can be obtained from the corresponding author upon request (Yunsheng Wang).
