# Supplementary material for: Functional analysis of the dihydroflavonol 4-reductase family of Camellia sinensis: exploiting key amino acids to reconstruct reduction activity
Source: Hortic Res. 2022 Apr 22;9:uhac098. doi: 10.1093/hr/uhac098 (PMC9250652; doi:10.1093/hr/uhac098)
Supplement: Web_Material_uhac098 [file web_material_uhac098.zip › Supplemental Data.doc]

**Figure S1. Schematic representation of *CsDFRs* gene structures and promoter elements.** (A) Electrophoresis diagram of CsDFRs genes. (B) Four DFR genes sequences were used in Gene Structure Display Server tool to determine gene structure, introns and exon indicated by the green box and black line. (C) PlantCARE database was used to analyze putative *cis*-acting elements in the promoter region of *CsDFR* genes. Numbers indicate the positions of the motifs. Motifs: MYBST1, GGATA; MYBPLANT, MACCWAMC; MYBPZM, CCWACC; E-BOX, CANNTG.

**Supplementary Table 1. Relative quantity of different flavonoid compounds in different tissues.**

| Compound | Bud | Young leaf | Mature leaf | Stem | Root |
| --- | --- | --- | --- | --- | --- |
| Flavan-3-ols (mg/g) | 125.73±4.92 c | 240.02±8.77 a | 209.91±4.14 b | 46.22±0.63 d | 2.86±0.06 e |
| Proanthocyanidins (mg/g) | 14.57±0.03 e | 25.11±0.07 c | 18.21±0.03 d | 28.35±0.35 b | 47.28±1.23 a |
| Flavonols (peak area) | 47163±1072 c | 465637±4055 a | 356266±2880 b | 9711±620 d | 763±45 e |

Note: The data were quoted from Jiang (Jiang *et al*., 2013). The different letters (a, b, c, d and e) indicated the significant level at P＜0.05.

**Supplementary Table 2. The primers used in this study**

| **Gene names** | **Sequence (5’→3’)** |
| --- | --- |
| **Primers for gene cloning** |  |
| *CsDFRa-F* | ATGAAAGACTCTGTTGC |
| *CsDFRa-R* | TTAAACCTTGTTGCCATTG |
| *CsDFRb1-F* | ATGGAAGTAGTGGAAG |
| *CsDFRb1-R* | CTATAGAGATGGCATCTG |
| *CsDFRb3-F* | ATGGAAGAAGAAAGAGAG |
| *CsDFRb3-R* | CTATAGTGTCAACCACC |
| *CsDFRc-F* | ATGGAAGATACAAAAAG |
| *CsDFRc-R* | CTAGTTTACAGTAGGAC |
| **Primers for qRT-PCR** |  |
| GAPDH-qRT-F | TTGGCATCGTTGAGGGTCT |
| GAPDH-qRT-R | CAGTGGGAACACGGAAAGC |
| *CsDFRa-qRT-F* | ATTCATCGGCTCGTGGC |
| *CsDFRa-qRT-R* | TTCTCAGGGTCCTTAGACTCAA |
| *CsDFRb1-qRT-F* | CTTCAATGGCACTTGCCTTAATCAC |
| *CsDFRb1-qRT-R* | TTGTACTCCGGGTATTTCTTGCTCA |
| *CsDFRb3-qRT-F* | ATGTGCCTGTTCTTCCTG |
| *CsDFRb3-qRT-R* | TCGATGCTGACAGGTTTCAA |
| *CsDFRc-qRT-F* | TCGTGTCTGGAAAACAAAAACAGGT |
| *CsDFRc-qRT-R* | AGAACTTGAACGCTTGTTGGAACTG |
| **Primers for SUMO vector recombinant** |  |
| *SUMO-CsDFRa-F* | CGCGAACAGATTGGAGGTATGAAAGACTCTGTTGC |
| *SUMO-CsDFRa-R* | GTGGCGGCCGCTCTATTATTAAACCTTGTTGCCATTG |
| *SUMO-CsDFRb1-F* | CGCGAACAGATTGGAGGTATGGAAGTAGTGGAAG |
| *SUMO-CsDFRb1-R* | GTGGCGGCCGCTCTATTACTATAGAGATGGCATCTG |
| *SUMO-CsDFRb3-F* | CGCGAACAGATTGGAGGTATGGAAGAAGAAAGAGAG |
| *SUMO-CsDFRb3-R* | GTGGCGGCCGCTCTATTACTATAGTGTCAACCACC |
| *SUMO-CsDFRc-F* | CGCGAACAGATTGGAGGTATGGAAGATACAAAAAG |
| *SUMO-CsDFRc-R* | GTGGCGGCCGCTCTATTACTAGTTTACAGTAGGAC |

**Supplementary Table 3. Kinetic parameters for DFRa, DFRc, DFRb1N120S and DFRb1C126T.**

| Enzyme | Substrate | *K_m_*(μM) | *K_cat_*(s^-1^) | *K_cat_*/*K_m_*(mM^-1^s^-1^) |
| --- | --- | --- | --- | --- |
| DFRa | DHK | 145.10±13.85 | 5.41±0.09 | 37.31 |
|  | DHQ | 41.80±4.60 | 10.39±0.88 | 248.56 |
|  | DHM | 58.44±13.85 | 10.47±0.18 | 179.87 |
|  |  |  |  |  |
| DFRc | DHK | 42.31±1.85 | 5.74±0.14 | 135.69 |
|  | DHQ | 81.80±5.19 | 7.21±0.22 | 88.14 |
|  | DHM | 105.56±44.85 | 15.24±3.83 | 145.14 |
|  |  |  |  |  |
| DFRb1N120S | DHK | 148.96±17.53 | 6.76±0.32 | 61.79 |
|  | DHQ | 108.66±1.91 | 10.97±0.17 | 115.89 |
|  | DHM | 96.58±4.37 | 8.51±0.20 | 57.43 |
|  |  |  |  |  |
| DFRb1C126T | DHK | 131.09±22.06 | 6.66±0.78 | 51.10 |
|  | DHQ | 102.57±4.55 | 6.66±0.17 | 64.92 |
|  | DHM | 47.75±13.01 | 8.95±1.15 | 184.14 |

Note:Two micrograms of purified recombinant proteins were used in each [enzyme](../../../../D:/QQ%25E9%259F%25B3%25E4%25B9%2590/Youdao/Dict/7.5.2.0/resultui/dict/%3Fkeyword=enzyme) [reaction](../../../../D:/QQ%25E9%259F%25B3%25E4%25B9%2590/Youdao/Dict/7.5.2.0/resultui/dict/%3Fkeyword=reaction) assay. A fixed concentration of 0.05mg dihydroflavonol (DHK, DHQ and DHM) was used as substrate. The data are presented as means±SD (n=3).
